# Post-traumatic stress disorder associated with life-threatening motor vehicle collisions in the WHO World Mental Health Surveys

**DOI:** 10.1186/s12888-016-0957-8

**Published:** 2016-07-22

**Authors:** Dan J. Stein, Elie G. Karam, Victoria Shahly, Eric D. Hill, Andrew King, Maria Petukhova, Lukoye Atwoli, Evelyn J. Bromet, Silvia Florescu, Josep Maria Haro, Hristo Hinkov, Aimee Karam, María Elena Medina-Mora, Fernando Navarro-Mateu, Marina Piazza, Arieh Shalev, Yolanda Torres, Alan M. Zaslavsky, Ronald C. Kessler

**Affiliations:** Dept of Psychiatry and Mental Health, University of Cape Town and Groote Schuur Hospital, Cape Town, South Africa; St George Hospital Medical Center, Balamand University, Faculty of Medicine, Institute for Development, Research, Advocacy & Applied Care, Beirut, Lebanon; Department of Health Care Policy, Harvard Medical School, Boston, USA; Department of Mental Health, Moi University School of Medicine, Eldoret, Kenya; Department of Psychiatry, Stony Brook University School of Medicine, Stony Brook, USA; National School of Public Health, Management and Professional Development, Bucharest, Romania; Parc Sanitari Sant Joan de Déu, Centros de Investigación Biomédica en Red de Salud Mental, Universitat de Barcelona, Barcelona, Spain; National Center for Public Health and Analyses, Sofia, Bulgaria; Institute for Development, Research, Advocacy & Applied Care (IDRAAC), Beirut, Lebanon; National Institute of Psychiatry Ramón de la Fuente, Mexico City, Mexico; Subdirección General de Salud Mental, Servicio Murciano de Salud, Instituto Murciano de Investigación Biosanitaria Virgen de la Arrixaca, Centro de Investigación Biomédica en Red de Epidemiología y Salud Pública, Murcia, Spain; National Institute of Health, Lima, Peru; Department of Psychiatry, New York University Langone Medical Center, New York, USA; Center for Excellence on Research in Mental Health, CES University, Medellin, Colombia

**Keywords:** Posttraumatic stress disorder, PTSD, Motor vehicle collision

## Abstract

**Background:**

Motor vehicle collisions (MVCs) are a substantial contributor to the global burden of disease and lead to subsequent post-traumatic stress disorder (PTSD). However, the relevant literature originates in only a few countries, and much remains unknown about MVC-related PTSD prevalence and predictors.

**Methods:**

Data come from the World Mental Health Survey Initiative, a coordinated series of community epidemiological surveys of mental disorders throughout the world. The subset of 13 surveys (5 in high income countries, 8 in middle or low income countries) with respondents reporting PTSD after life-threatening MVCs are considered here. Six classes of predictors were assessed: socio-demographics, characteristics of the MVC, childhood family adversities, MVCs, other traumatic experiences, and respondent history of prior mental disorders. Logistic regression was used to examine predictors of PTSD. Mental disorders were assessed with the fully-structured Composite International Diagnostic Interview using DSM-IV criteria.

**Results:**

Prevalence of PTSD associated with MVCs perceived to be life-threatening was 2.5 % overall and did not vary significantly across countries. PTSD was significantly associated with low respondent education, someone dying in the MVC, the respondent or someone else being seriously injured, childhood family adversities, prior MVCs (but not other traumatic experiences), and number of prior anxiety disorders. The final model was significantly predictive of PTSD, with 32 % of all PTSD occurring among the 5 % of respondents classified by the model as having highest PTSD risk.

**Conclusion:**

Although PTSD is a relatively rare outcome of life-threatening MVCs, a substantial minority of PTSD cases occur among the relatively small proportion of people with highest predicted risk. This raises the question whether MVC-related PTSD could be reduced with preventive interventions targeted to high-risk survivors using models based on predictors assessed in the immediate aftermath of the MVCs.

**Electronic supplementary material:**

The online version of this article (doi:10.1186/s12888-016-0957-8) contains supplementary material, which is available to authorized users.

## Background

Motor vehicle collisions (MVCs) are a substantial contributor to the global burden of disease, with most recent estimates putting them as the 5^th^ leading contributor to disability adjusted life years worldwide [1]. A growing literature has investigated the relationship between MVCs and posttraumatic stress disorder (PTSD) based on evidence that MVC survivors recruited from emergency units and hospital wards have high PTSD prevalence [2–5]. Community surveys find lower PTSD prevalence [2–5] and suggest that risk of PTSD is considerably more common after traumatic experiences involving interpersonal violence [6, 7]. However, even a relatively low prevalence of MVC-related PTSD would represent a significant global public health problem given the enormous number of MVCs that occur across the world each year.

Many questions about MVC-related PTSD remain unaddressed. Perhaps the most fundamental of these is that the vast majority of existing studies examining MVC-related PTSD come from a few high-income countries, making it unclear whether similar findings hold elsewhere. This is an especially important limitation given that 90 % of traffic deaths occur in low- and middle-income countries, with fatality rates more than twice as high in these regions as in high-income countries [8].

We address this limitation in the current report by analyzing data on prevalence and predictors of MVC-related PTSD from community epidemiological surveys carried out in 8 low-middle income countries and 4 high income countries in the WHO World Mental Health Survey Initiative (www.hcp.med.harvard.edu/wmh). The predictors we focus on are consistent with those examined and found to be significant in previous community epidemiological surveys of MVC-related PTSD [9–11] and overall PTSD [12–14]: socio-demographics, characteristics of the trauma; prior history of exposure to other highly stressful experiences; and history of prior psychopathology.

## Methods

### Samples

The World Mental Health surveys are a series of cross-national community epidemiological surveys using consistent sampling, field procedures, and instruments designed to facilitate pooled cross-national analyses of prevalence and correlates of common mental disorders [15]. The data reported here come from a subset of 13 World Mental Health surveys that used an expanded assessment of PTSD (described below) with a sufficient number of respondents reporting life-threatening MVCs to observe at least one case of associated PTSD. The surveys included 5 in countries classified by the World Bank [16] as high income countries (national surveys in Germany, Israel, Spain, and the United States along with a regional survey in Spain [Murcia]) and 8 in countries classified as low- or middle-income countries (national surveys in Bulgaria, Lebanon, Mexico, Peru, Romania, South Africa, and Ukraine along with a regional survey in Colombia [Medellin]). Each survey was based on a probability sample of household residents in the target population using a multi-stage clustered area probability sample design. Response rates ranged from 57.8 % (Germany) to 97.2 % (Colombia) and had a weighted mean of 75.0 % across surveys. A more detailed description of sampling procedures is presented elsewhere [17]. (See Additional file 1: Table S1)

### Field procedures

Interviews were administered face-to-face in respondent homes after obtaining informed consent using procedures approved by local Institutional Review Boards. The interview schedule was developed in English and translated into other languages using a standardized WHO translation, back-translation, and harmonization protocol [18]. Bilingual supervisors from each country were trained and supervised by the World Mental Health Survey Initiative Data Collection Coordination Centre to guarantee cross-national consistency in field procedures [18].

Interviews were in two parts. Part I, administered to all respondents, assessed core DSM-IV mental disorders (*n* = 58,335 respondents across all surveys). Part II assessed additional disorders and correlates. Questions about traumatic experiences and PTSD were included in Part II, which was administered to 100 % of Part I respondents who met lifetime criteria for any Part I disorder and a probability subsample of other Part I respondents (*n* = 32,946). Part II respondents were weighted to adjust for differential within and between household selection, selection into Part II, and deviations between the sample and population demographic-geographic distributions. More details about sample design and weighting are presented elsewhere [17].

### Measures

#### Traumatic experiences

The interview asked about lifetime exposure to each of 27 different types of traumatic experiences in addition to two open-ended question about exposure to *“any other”* traumatic experience and to a *private* experience the respondent did not want to name. Given that many respondents reported exposure to multiple traumatic experiences, it was impossible to ask about the details of each one. We consequently evaluated PTSD associated with the self-reported worst lifetime traumatic experience reported by each respondent as well as for one computer-generated randomly-selected traumatic experience from all ever experienced by the respondent. In cases where the respondent only experienced one occurrence of one traumatic experience in his or her lifetime, that single occurrence was the only one evaluated for PTSD. In cases where distinct worst and randomly-selected traumatic experiences were considered, we checked for overlap (e.g., an unexpected death of a loved one that occurred during a respondent’s MVC), respondents were probed for overlap between the two experiences. When overlap was found, PTSD was assessed only once for the overall situation and no attempt was made to determine whether the PTSD was due to one, the other, or some combination of the components of the overall situation.

#### Characteristics of MVCs perceived as life-threatening

One of the experiences assessed was a “life-threatening” motor vehicle collision. The term *life-threatening* was not further specified, but was included in the description to minimize reports of minor accidents. Four questions were asked about these randomly-selected MVCs that might have influenced likelihood of PTSD: whether the respondent was the driver or passenger; the respondent’s judgment about whether the MVC was caused by the respondent, someone else in the respondent’s vehicle, someone not in the respondent’s vehicle, and/or situational factors such as hazardous driving conditions or a mechanical error; whether anyone was killed and the relationship of that/those person(s) to the respondent; and whether anyone (including the respondent) was seriously injured and the relationship of that/those person(s) to the respondent. We also asked whether the respondent was a pedestrian, cyclist, or bystander, but these circumstances were seldom reported and were invariably not associated with PTSD. We consequently focused analysis on cases where the respondent was either a driver or passenger in a motor vehicle.

#### PTSD

Mental disorders were assessed with the Composite International Diagnostic Interview [19], a fully-structured interview administered by trained lay interviewers, to assess DSM-IV and ICD-10 disorders. DSM-IV criteria are used here. As detailed elsewhere [20], blinded clinical reappraisal interviews with the Structured Clinical Interview for DSM-IV conducted in four countries found concordance for DSM-IV PTSD to be moderate [21] (AUC = .69). Sensitivity and specificity were .38 and .99, respectively, resulting in a likelihood ratio positive of 42.0, which is well above the threshold of 10 typically used to consider screening scale diagnoses definitive [22]. Consistent with this high value, the proportion of estimated cases confirmed by the Structured Clinical Interview for DSM-IV was 86.1 %, suggesting that the vast majority of respondents classified as having PTSD would independently have been judged to have PTSD by a trained clinician.

#### Other mental disorders

The diagnostic interview also assessed three lifetime DSM-IV mood disorders (major depressive disorder, dysthymic disorder, and broadly-defined bipolar disorder [including bipolar I and II and subthreshold bipolar disorder, which was defined using criteria described elsewhere [23]), six lifetime anxiety disorders (panic disorder with or without agoraphobia, agoraphobia without a history of panic disorder, specific phobia, social phobia, generalized anxiety disorder, prior [to the randomly selected traumatic experience] posttraumatic stress disorder, and separation anxiety disorder), four disruptive behaviour disorders (attention-deficit/hyperactivity disorder, oppositional-defiant disorder, conduct disorder, and intermittent explosive disorder), and two substance disorders (alcohol abuse with or without dependence; drug abuse with or without dependence). Age-of-onset of each disorder was assessed using special probing techniques shown experimentally to improve recall accuracy [24]. This allowed us to determine based on retrospective age-of-onset reports whether each respondent had a history of each disorder prior to the age of occurrence of the randomly selected traumatic experience. DSM-IV organic exclusion rules and diagnostic hierarchy rules were used (other than for oppositional defiant disorder, which was defined with or without conduct disorder, and substance abuse, which was defined with or without dependence). Agoraphobia was combined with panic disorder because of low prevalence. Dysthymic disorder was combined with major depressive disorder for the same reason. These aggregations resulted in information being available on 14 prior lifetime disorders (one of which was prior PTSD). As detailed elsewhere [20], generally good concordance was found between these estimated diagnoses and blinded clinical diagnoses based on reappraisal interviews [25].

#### Other predictors of PTSD

We examined six classes of predictors. The first two were described above: the characteristics of the MVC and the respondent’s history of prior mental disorders. The third were socio-demographics, which included age, education, and marital status, each defined as of the time of the randomly selected traumatic experience, and gender. Given the wide variation in education levels across countries, education was classified as high, high-average, low-average, or low according to within-country norms. Details on this coding scheme are described elsewhere [26]. The next three classes of predictors assessed the respondent’s history of exposure to stressful experience prior to the randomly selected MVC: whether the respondent had been in one or more previous MVCs perceived as life-threatening; exposure to other lifetime traumatic experiences from the set of 29 assessed in the surveys; and exposure to each of 12 childhood family adversities. Consistent with prior research on childhood adversities [27], we distinguished between those in a highly-correlated set of 7 that we labelled Maladaptive Family Functioning adversities (parental mental disorder, parental substance abuse, parental criminality, family violence, physical abuse, sexual abuse, neglect) and other adversities (parental divorce, parental death, other parental loss, serious physical illness, family economic adversity). Details on the measurement of childhood adversities are presented elsewhere [27].

### Analysis methods

In addition to the sample weight described above in the subsection on samples, each respondent who reported traumatic experiences was weighted by the inverse of the probability of selection of the specific occurrence assessed. For example, a respondent who reported 3 traumatic experience types and 2 occurrences of the randomly selected type would receive a traumatic experience weight of 6.0. The product of this with the Part II weight was used in analyses of the randomly selected traumatic experiences, yielding a sample that is representative of all lifetime traumatic experiences occurring to all respondents. As respondents with a randomly-selected MVC perceived as life-threatening are the focus of this report, the sum of the consolidated weights across this subset of respondents was standardized in each country for purposes of pooled cross-national analysis to equal the observed number of respondents with randomly-selected MVCs.

Logistic regression was used to examine predictors of PTSD pooled across surveys. Predictors were entered in blocks, beginning with socio-demographics (Model 1), followed by MVC characteristics (Model 2), prior stressor exposure (Model 3), and prior mental disorders (Model 4). This sequence was used to allow us to examine the extent to which the significant coefficients in earlier models were explained statistically by predictors introduced in later models. All models included dummy control variables for surveys, which means coefficients represent pooled within-survey coefficients. Logistic regression coefficients and standard errors were exponentiated and are reported as odds-ratios (ORs) with 95 % confidence intervals (CIs). Statistical significance was evaluated using .05-level two-sided tests. The design-based Taylor series method [28] implemented in the SAS software system [29] was used to adjust for the weighting and clustering of observations. Design-based F tests were used to evaluate the significance of predictor sets, with numerator degrees of freedom equal to the number of variables in the set and denominator degrees of freedom equal to the number of geographically-clustered sampling error calculation units containing randomly-selected MVCs across surveys (*n* = 416) minus the sum of primary sample units from which these sampling error calculation units were selected (*n* = 284) and one less than the number of variables in the predictor set [30].

Once the final model was estimated, a predicted probability of PTSD was generated for each respondent from model coefficients. A receiver operating characteristic curve was then calculated from this summary predicted probability [31]. Area under the curve (AUC) was calculated to quantify overall prediction accuracy [32]. We also evaluated sensitivity and positive predictive value of the model among the 5 % of respondents with highest predicted probabilities to determine how well the model implies subsequent PTSD could be predicted if the model was applied as part of a screening effort in the immediate aftermath of the MVC. Sensitivity is the proportion of observed PTSD cases found among the 5 % of respondents with highest predicted probabilities. Positive predictive value is the prevalence of PTSD among this 5 % of respondents. We used the method of replicated 10-fold cross-validation with 20 replicates (i.e., 200 separate estimates of model coefficients) to correct for the over-estimation of prediction accuracy when estimating and evaluating model fit in the same sample [33].

## Results

### Exposure to traumatic experiences

A weighted 69.1 % of Part II respondents across surveys reported lifetime exposure to at least one traumatic experience (Table 1). One-fourth (24.6 %) of these respondents reported only one occurrence, while the others reported a mean of 6.0 occurrences (range 2–160; inter-quartile range 3–6). MVCs perceived as life-threatening represented a weighted 14.3 % of these occurrences, making them the fourth most common traumatic experience, exceeded only by unexpected death of a loved one, being mugged, and witnessing a serious injury or death, (More detailed information on distributions of traumatic experienced are presented in Additional file 1: Table S2) A MVC considered by the respondent as life-threatening was the randomly selected traumatic experience for 649 respondents across the 13 surveys, ranging from a low of 17 in Lebanon to a high of 168 in the United States. As some indication of the severity of these MVCs, someone died or was seriously injured in 41.8 % of those MVCs in high income countries and 38.1 % in low or middle income countries.

**Table 1:** Distribution of lifetime exposure to traumatic experiences and motor vehicle collisions (MVCs) in the participating World Mental Health surveys

	Proportion of respondents exposed to any lifetime traumatic experience		Proportion of respondents exposed to any lifetime MVC perceived as life-threatening		Mean number of reported MVCs among those with any		Reported MVCs as a proportion of all lifetime traumatic experiences		(n) of respondents with randomly selected MVCs^a^
	%	(SE)	%	(SE)	Mean	(SE)	%	(SE)	
High									
Germany	67.3	(2.2)	9.1	(1.1)	1.1	(0.0)	3.8	(0.6)	(20)
Israel	74.8	(0.7)	11.6	(0.5)	1.3	(0.0)	4.7	(0.2)	(35)
Spain	54.0	(1.7)	14.1	(1.2)	1.3	(0.0)	12.3	(1.1)	(58)
Spain (Murcia)	62.4	(1.9)	11.9	(0.7)	1.3	(0.1)	9.6	(1.1)	(39)
United States	82.7	(0.9)	19.2	(0.9)	1.5	(0.0)	6.0	(0.2)	(168)
Total	73.0	(0.6)	14.6	(0.4)	1.4	(0.0)	6.0	(0.1)	(320)
Low or middle									
Bulgaria	28.6	(1.3)	6.9	(0.9)	1.4	(0.1)	12.8	(1.4)	(31)
Lebanon	81.1	(2.7)	12.8	(1.4)	1.3	(0.1)	4.4	(0.5)	(17)
Colombia (Medellin)	75.1	(2.6)	18.4	(1.7)	1.4	(0.1)	6.5	(0.6)	(52)
Mexico	68.8	(1.8)	15.3	(1.2)	1.3	(0.0)	6.8	(0.5)	(42)
Peru	83.1	(0.8)	20.1	(1.2)	1.4	(0.0)	7.4	(0.4)	(34)
Romania	41.5	(1.1)	9.3	(0.6)	1.4	(0.1)	10.2	(0.8)	(63)
South Africa	73.8	(1.2)	13.2	(0.6)	1.2	(0.0)	5.1	(0.2)	(52)
Ukraine	84.6	(1.7)	21.1	(1.3)	1.3	(0.0)	7.3	(0.6)	(38)
Total	65.6	(0.6)	14.1	(0.4)	1.3	(0.0)	6.6	(0.2)	(329)
Total	69.1	(0.4)	14.3	(0.3)	1.4	(0.0)	6.3	(0.1)	(649)

^a^The surveys considered here are limited to the subset of World Mental Health surveys that obtained information about TE characteristics associated with one randomly selected lifetime traumatic experience for each respondent and in which a sufficient number of respondents with a randomly selected MVC was included for at least one such respondent to have met DSM-IV/CIDI criteria for PTSD associated with that MVC

### Socio-demographic distribution of MVCs perceived as life-threatening

The majority of MVCs perceived as life-threatening occurred to men (59.1 %), a plurality (42.8 %) during young adulthood (ages 18–29), and the vast majority of others during either childhood (11.7 %; ages 1–12), adolescence (15.8 %; ages 13–17), or middle age (24.7 %; ages 30–44). (Distributions of all predictors are presented in Additional file 1: Table S3) Consistent with this age distribution, 66.9 % of reported MVCs occurred to people who had never been married at the time of the MVC (25.1 % currently married, 8.0 % previously married). A higher proportion occurred to people with high-average (38.1 %) than low (27.9 %), low-average (20.5 %), or high (13.5 %) education.

### Prevalence of PTSD associated with MVCs perceived as life-threatening

Prevalence of MVC-related PTSD averaged 2.8 % across the surveys that had at least one case of MVC-related PTSD, but was 2.5 % when we also included surveys with no cases of MVC-related PTSD (Table 2). A total of 36 respondents across surveys had PTSD associated with their randomly-selected MVC. Prevalence did not vary significantly either over the full set of surveys with any cases (χ^2^_12_ = 8.5, *p* = .75), between surveys in high and low-middle income countries (χ^2^_1_ = 0.1, *p* = .71), within surveys in high income countries (χ^2^_4_ = 3.7, *p* = .06), or within surveys in low-middle income countries (χ^2^_7_ = 4.7, *p* = .69). It is noteworthy that the eight surveys that were excluded from further analysis because no cases of PTSD were observed (national surveys in Belgium, Colombia, France, Italy, Netherlands, and Northern Ireland; regional surveys in Brazil and Japan) had numbers of respondents with randomly-selected MVCs (*n* = 9–46) small enough that the 95 % confidence interval of PTSD prevalence after the MVC based on the Wilson method (a method that allow the confidence interval to be calculated for a sample prevalence of 0.0 %; [34]) in these surveys included the 2.8 % prevalence observed in the surveys included in the analysis.

**Table 2:** Prevalence of DSM-IV/CIDI PTSD after randomly selected MVCs perceived as life-threatening in the participating World Mental Health surveys^a^

	% PTSD	(95 % CI)^b^	Number with PTSD (n)	Total sample size (n)
High income countries				
Germany	1.0	(0.0–3.3)	(1)	(20)
Israel	5.5	(0.0–12.8)	(3)	(35)
Spain	1.2	(0.0–3.2)	(4)	(58)
Spain – Murcia	0.2	(0.0–0.6)	(1)	(39)
United States	4.2	(0.0–8.5)	(9)	(168)
Total	3.1	(0.7–5.6)	(18)	(320)
χ^2^_4_^c^		3.7		
Low or middle income countries				
Bulgaria	6.7	(0.0–14.3)	(5)	(31)
Lebanon	2.0	(0.0–6.1)	(2)	(17)
Colombia – Medellin	1.8	(0.0–4.6)	(2)	(52)
Mexico	0.7	(0.0–1.9)	(2)	(42)
Peru	0.5	(0.0–1.6)	(1)	(34)
Romania	1.8	(0.0–5.3)	(1)	(63)
South Africa	5.6	(0.0–13.9)	(2)	(52)
Ukraine	1.5	(0.0–4.4)	(2)	(38)
Total	2.6	(0.8–4.3)	(17)	(329)
χ ^2^_7_^c^		4.7		
Total	2.8	(1.3–4.3)	(35)	(649)
χ ^2^_12_^c^		8.5		
χ ^2^_1_^d^		0.1		

^a^All results are based on weighted data that adjust for between-person differences in number of lifetime traumatic experiences within each survey. World Mental Health surveys that had too few randomly selected MVCs (numbers of such cases are reported in parentheses) for any to meet criteria for PTSD were excluded. These surveys were those in Brazil (23), Colombia (25), Japan (25), Northern Ireland (25), Belgium (13), France (34), Italy (46), Netherlands (9)

^b^The Wilson interval method [34] was used to calculate confidence intervals when the lower bound was less than 0.0.

^c^These *χ* ^2^ tests evaluate the significance of between-survey difference in prevalence among surveys in high income countries (*p* = 0.45), among surveys in low-middle income countries (*p* = 0.69), and across all 13 surveys (*p* = 0.71)

^d^This *χ* ^2^ test evaluates the significance of between-survey difference in prevalence between surveys in high and low-middle income countries (*p* = 0.71)

### Predictors of PTSD

#### Models 1 and 2

Respondent education was significantly and inversely related to odds of PTSD in the wake of a life-threatening MVC (Model 1: OR 0.6; 95 % CI 0.5–0.8), while the other socio-demographic characteristics considered (i.e., respondent age and marital status at the time of the MVC and sex) were not significant predictors (Table 3). The OR of being married at the time of the MVC was large in substantive terms (5.7), but was not significant due to the low proportion of respondents who were married at the time of their MVC and the rarity of PTSD. (Prevalence of each predictor and the bivariate association of each predictor with PTSD are presented in Additional file 1: Table S3). The design effects introduced by the weights also contributed to the wide CI of the OR for being married, although age, sex, and marital status remained insignificant when the model was estimated again using unweighted data and a statistical control for the TE-level weight.

**Table 3:** Predictors of DSM-IV/CIDI PTSD among World Mental Health survey respondents after randomly selected MVCs perceived as life threatening (*n* = 649)^b^

	Model 1		Model 2		Model 3		Model 4	
	OR	(95 % CI)	OR	(95 % CI)	OR	(95 % CI)	OR	(95 % CI)
I. Socio-demographics								
Age in decades	1.4	(0.9–2.4)	1.5	(0.8–2.6)	2.1^a^	(1.3–3.3)	2.2^a^	(1.3–3.6)
Male (vs. female)	0.6	(0.3–1.4)	0.6	(0.2–1.4)	0.7	(0.3–1.6)	1.2	(0.4–3.3)
Education^d^	0.6^a^	(0.5–0.8)	0.6^a^	(0.4–0.9)	0.7	(0.5–1.1)	0.7	(0.5–1.1)
Currently (vs. never) married	5.7	(0.5–60.0)	6.0	(0.7–49.7)	4.8	(1.0–23.3)	2.4	(0.8–7.6)
Previously (vs. never) married	1.4	(0.2–7.9)	2.1	(0.3–14.7)	1.8	(0.3–12.0)	1.6	(0.3–9.3)
F_2,131_^c^	1.3		1.4		1.8		1.2	
II. Trauma characteristics								
R was the driver (vs. passenger)^e^	--	--	2.3	(0.7–7.2)	1.8	(0.6–5.2)	1.0	(0.3–3.8)
Fault of someone else (vs respondent)	--	--	2.7	(0.8–9.4)	2.3	(0.8–6.9)	2.2	(0.8–6.1)
No fault (vs. respondent)	--	--	3.7	(0.8–17.8)	3.9	(0.9–16.1)	2.3	(0.7–7.2)
F_2,131_^c^			1.6		2.5		1.3	
Someone died	--	--	7.7^a^	(4.4–13.4)	12.6^a^	(6.4–24.8)	9.9^a^	(4.4–22.2)
Respondent seriously injured	--	--	3.5^a^	(1.4–8.8)	3.1^a^	(1.0–9.0)	2.9^a^	(1.0–8.5)
Someone else seriously injured	--	--	3.1^a^	(1.5–6.3)	3.1^a^	(1.5–6.3)	3.9^a^	(1.9–8.0)
III. Prior vulnerability factors								
Prior MVCs (0-2+)	--	--	--	--	3.2^a^	(1.4–7.7)	5.1^a^	(1.6–15.9)
Childhood adversities^f^	--	--	--	--	10.2^a^	(2.2–47.5)	3.6	(0.6–20.4)
Number of prior anxiety disorders (0-3+)	--	--	--	--	--	--	4.7^a^	(2.5–8.9)
F_(5, 11,,13,14), (128, 122, 121, 120)_^c^	11.0^a^		15.2^a^		16.6^a^		13.7^a^	

^a^Significant at the .05 level, two-sided test

^b^Based on pooled logistic regression models with 12 dummy variable controls for the 13 surveys. Regression models were weighted and controls were included for survey

^c^The design-based F tests evaluated the significance of predictor sets, with numerator degrees of freedom equal to the number of predictors in the set and denominator degrees of freedom equal to the number of geographically-clustered sampling error calculation units across surveys (*n* = 416) minus the sum of the number of primary sampling units across surveys (*n* = 284) and one minus the number of variables in the predictor set [30]

^d^Values for education ranged from 1 to 4 (low, low-average, high-average, and high)

^e^The analysis was limited to respondents who were either drivers or passengers

^f^A dummy variable for 2+ Maladaptive Family Functioning childhood adversities

Three MVC characteristics were positively and significantly associated with PTSD in Model 2: someone dying in the collision (which occurred in 4.9 % of MVCs; OR 7.7; 95 % CI 4.4–13.4), the respondent being seriously injured (which occurred in 31.4 % of MVCs; OR 3.5; 95 % CI 1.4–8.8), and someone else being seriously injured (which occurred in 20.5 % of MVCs; OR 3.1; 95 % CI 1.5–6.3). The numbers of other people who died or were seriously injured were too small to distinguish effects depending on relationship with the respondent. The other MVC characteristics -- whether the respondent was the driver (45.9 % of MVCs) or a passenger, and perceived fault (20.6 % respondent, 62.8 % someone else, 16.6 % circumstantial) -- were not significant predictors.

#### Model 3

Controlling for the predictors in Model 2, a history of prior MVCs (19.3 % of MVCs) was significantly associated with increased odds of PTSD (OR 3.2; 95 % CI 1.4–7.7). In comparison, preliminary analysis found that prior exposure to other traumatic experiences (55.6 % of all MVCs) was not associated consistently with PTSD either when we considered the 20 prior traumatic experiences (other than prior MVCs) found to be associated with at least one case of PTSD in a multivariate equation or when we created an aggregate measure of number of such prior experiences (0, 1, 2, 3+). (Detailed results are presented in Additional file 1: Table S4).

As noted in the section on measures, the World Mental Health surveys assessed 7 highly correlated childhood adversities that we labelled Maladaptive Family Functioning adversities. Five other childhood adversities were also assessed. Preliminary analyses showed that even though neither of these two sets of childhood adversities predicted PTSD significantly when considered together, a summary measure of number of maladaptive family functioning childhood adversities had a significant and positive dose–response relationship with PTSD due to a very high OR for respondents who experienced 2+ such adversities (14.7 % of all respondents). The OR for this measure of Maladaptive Family Functioning childhood adversities in Model 3 is 10.2 (95 % CI 2.2–47.5). (Detailed results of preliminary analyses are presented in Additional file 1: Table S5) One other noteworthy feature of Model is that the insignificant association between respondent age and PTSD in Models 1 and 2 became significantly elevated in Model 3 (OR 2.1; 95 % CI 1.3–3.3). More detailed analysis showed that this occurred because age was inversely related to maladaptive family functioning childhood adversities, leading to a suppression of the significantly positive direct association of age with PTSD in Model 3.

#### Model 4

Preliminary analyses showed found that half of the 14 prior mental disorders assessed in the surveys were associated with significantly elevated odds of PTSD after a MVC in models that added only one prior mental disorder at a time to the predictors in Model 3. (Detailed results are presented in Additional file 1: Table S6) Four of these 7 were anxiety disorders (specific and social phobia, generalized anxiety disorder, prior PTSD). Two were disruptive behavior disorders (ADHD, oppositional-defiant disorder). And the other was major depression/dysthymia. These significant ORs were in the range 5.8–57.1, but had wide confidence intervals due to the rarity of the individual disorders (which were present in 1.6–7.5 % of respondents prior to their randomly-selected MVC).

High comorbidity among these mental disorders resulted in the joint predictive associations of the 12 disorders other than conduct disorder and alcohol abuse being insignificant despite many of the individual disorders being significant when considered one at a time. However, a reduced model that included four summary measures of major depression, number of anxiety disorders, number of disruptive behavior disorders other than conduct disorder, and drug abuse found significant joint associations due to a significant OR of number of anxiety disorders in conjunction with insignificant ORs of the other three summary disorder measures. (Detailed results are presented in Additional file 1: Table S6) Based on this result, the only measure of prior mental disorders included in Model 4 was number of anxiety disorders. Each additional anxiety disorder in the range 0–3 (the maximum number of prior anxiety disorders with sufficient numbers of respondents in the sample to estimate a stable OR) was associated with an incremental elevated OR of 4.7 (CI 2.5–8.9). It is noteworthy that the introduction of prior anxiety disorders into the model led to the coefficient for childhood adversities becoming insignificant.

### Consistency and strength of overall model predictions

**Fig. 1:**
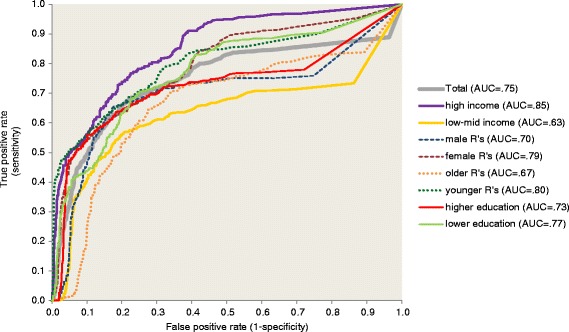
Area under the receiver operating characteristic curve for the final model (Model 4 in Table 3) in the total sample and selected subsamples. Note. "Older Rs" = 30+ years old; "Younger Rs" < 30 years old. "Higher education" = high and high-average; "Lower education" = low and low-average"

Although the small sample sizes precluded estimating model coefficients separately in each survey, we were able to compare overall model fit in surveys carried out in high income versus low-middle income countries by generating individual-level predicted probabilities based on Model 4 in the total sample and subsamples defined by country income level and respondent sex, age, and education. As noted in the section on analysis methods, the model was estimated 200 times using 20 replicates of 10-fold cross-validation in order to adjust estimates of model fit for the optimistic bias that exists when estimating model coefficients and evaluating model fit in the same dataset [33]. Estimated AUC based on this method was .75 in the total sample and .63–.85 in the subsamples (Fig. 1), all of which represent intermediate levels of classification accuracy [35]. The 5 % of respondents with highest predicted probabilities of suicide included 32.0 % of all cases of MVC-related PTSD (sensitivity) in the total sample, which is six times the concentration of risk expected by chance. Subgroup sensitivities range from a high of 49.8 % in high income countries to a low of 8.5 % among men (Table 4). Positive predictive value (the proportion of predicted positives who met criteria for PTSD) among the 5 % of respondents with higher predicted probabilities was 15.7 % in the total sample and ranged from a high of 26.4 % among females to a low of 3.7 % among males.

**Table 4:** Sensitivity and positive predictive value of a dichotomous classification distinguishing the 5 % of respondents with highest predicted probabilities of PTSD from other respondents based on replicated 10-fold cross-validation of the final model with 20 replications^a^

	Sensitivity^b^		Positive predictive value^c^	
	%PTSD	(SE)	%PTSD	(SE)
Total	32.0	(3.2)	15.7	(2.0)
Country income				
High	49.8	(4.4)	24.2	(3.4)
Low or middle	10.7	(2.4)	5.3	(1.3)
Age at collision				
30+ years old	17.7	(2.6)	8.7	(1.4)
< 30 years old	47.7	(4.9)	23.4	(3.8)
Sex				
Female	48.5	(4.1)	26.4	(3.3)
Male	8.5	(2.5)	3.7	(1.1)
Education				
Low or low-average	41.7	(4.1)	19.1	(2.7)
High or high-average	14.2	(3.1)	8.1	(1.9)

^a^Ten-fold cross-validation involves dividing the sample into 10 separate random subsamples of equal size, estimating the model in each of the 10 separate 90 % subsamples created by deleting 1 of the 10 subsamples, and applying predicted values based on each set of coefficients only to the remaining 10 % of the sample. Replicated cross-validation involves repeating the cross-validation process some number of times (20 times in the current application), with a different random split of the sample into 10 equal-sized subsamples each time. Sensitivity and positive predictive value were calculated separately in each of these 200 subsamples and averaged to produce the results reported here

^b^Sensitivity = Proportion of all PTSD found among the 5 % of respondent with highest predicted probabilities based on the final model

^c^Positive Predictive Value = Prevalence of PTSD among respondents in the row who are among the 5 % in the total sample with the highest predicted probabilities based on the final model

## Discussion

Four limitations are noteworthy. First, traumatic experiences and mental disorders were assessed retrospectively. Although the World Mental Health survey assessment procedure used a probing strategy shown experimentally to improve accuracy of timing estimates [24] and prospective evidence suggests that retrospective reports of traumatic experiences are valid [36], respondents with PTSD might nonetheless have been biased towards higher recall than other respondents of prior lifetime exposures and/or mental disorders [37–39]. Second, diagnoses were based on a fully-structured lay-administered interview rather than a semi-structured clinical interview. While the World Mental Health survey clinical appraisal data are reassuring [20], such work was undertaken in only a minority of countries. Third, the definition of a “life-threatening” MVC was left up to the respondent. Although it has been suggested that even minor injuries after an MVC can have significant sequelae [4, 40], we were unable to investigate this threshold issue because of this imprecision. Nor were we able to examine other psychopathological consequences of MVCs due to the fact that PTSD was the only mental disorder assessed in relation to randomly selected MVCs.

The most serious limitation of the study, though, involves the small number of respondents with PTSD (*n* = 36) in relation to the 14 predictors in the final model. The resulting 2.6 events-per-variable (EPV) ratio is well below the EPV of 10 often recommended as the minimum to avoid biased OR estimates [41]. Even though more recent statistical evaluations show that this rule often can be relaxed [42] and that other considerations (e.g., number of predictors, correlations among predictors, clustering, magnitudes of regression coefficients, selection rules used in building the model) are in many cases more important than EPV in determining model performance [43–45], caution is nonetheless needed in interpreting model results because of the low EPV.

Despite these limitations, our results are valuable in providing the first large-scale cross-national data on prevalence and predictors of MVC-related PTSD. Three results are particularly noteworthy. First, we found consistent evidence across surveys from different regions of the globe that PTSD is a relatively rare outcome of life-threatening MVCs. Pooled prevalence was 2.8 % in the surveys with at least one case of PTSD and 2.5 % when including surveys with no cases of PTSD. We are aware of only one previously-published study that reported a prevalence estimate that could be compared to ours: the Detroit Area Survey of Trauma in the United States, which, like the World Mental Health surveys, investigated PTSD associated with randomly-selected traumatic experiences. Conditional risk of PSTD after an MVC in that study was 2.3 % [40], an estimate quite similar to the 2.5–2.8 % in the current study. This focus on randomly selected traumatic experiences is important because more typical studies of PTSD after MVCs are based on unrepresentative samples of people who present to hospital emergency departments, over-representing MVC survivors who either had serious injuries or had high anxiety in the aftermath of their accidents [2–5]. It is also noteworthy that PTSD prevalence estimates associated with a number of other traumatic experiences in the World Mental Health surveys, most notably those involving interpersonal violence, were considerably higher than the prevalence estimate for PTSD after MVCs (e.g., 19.0 % prevalence of PTSD associated with rape and 11.7 % with intimate partner violence) [7].

Second, the significant predictors found here were generally consistent with previous research. Exclusion from the analysis of surveys with no cases of MVC-related PTSD did not bias these results, as data from such surveys would not have contributed to estimates of pooled within-survey ORs. Our finding that low education was the only significant predictor of MVC-related PTSD is broadly consistent with a meta-analysis that found low education to be the most consistent socio-demographic predictor of all forms of PTSD [12], although a more recent systematic review focused specifically on PTSD after MVCs found no consistent socio-demographic predictors [4]. Our findings that death and serious injury predicted MVC-related PTSD are consistent with findings from both clinical [4] and community epidemiological [9] studies. Our findings that being the driver and perceived fault were not significant predictors are consistent with a systematic review of previous studies of MVC-related PTSD [4], although other work on PTSD has found an association between trauma perpetration and PSTD [46], suggesting that more detailed study of the role of post-traumatic cognitions in MVC-related PTSD is needed [10].

Previous studies of MVC-related PTSD are not consistent with our findings that history of MVCs is a significant predictor of elevated PTSD risk while history of other traumatic experiences is not a significant predictor [2]. The broader literature has generally found that history of traumatic experiences is associated with increased PTSD risk after subsequent re-traumatization [7, 12, 14]. Caution is consequently needed in interpreting our negative result in this regard. If true, though, our result regarding the lack of an association between history of exposure to traumatic experiences other than MVCs and MVC-related PTSD might mean that the trauma “sensitization” or “scarring” associated with MVCs is specific [47], although another possibility is that the individuals involved in multiple MVCs have other vulnerability factors not captured in the measures of vulnerability included in our model [48, 49]. In comparison, evidence from previous studies is consistent with our finding that childhood adversities predict PTSD among individuals exposed to traumatic experiences [12, 50], although the specificity of this relationship may be low [27]. It is notable that in the current study, while childhood adversities were associated with MVC-related PTSD, intervening anxiety disorders partially mediated this association.

While we found that several prior mental disorders significantly predicted MVC-related PTSD, high comorbidities made it impossible to pinpoint specific disorders as being most important, resulting in the final best-fitting model including a count of anxiety disorders (including prior PTSD) as the measure of prior psychopathology. While meta-analyses of the broad PTSD literature have found that many prior mental disorders predict PTSD [12, 13], a systematic review of MVC-related PTSD showed that anxiety disorders were especially important predictors [4]. It has been suggested that anxious drivers engage in behaviors that increase risk of MVCs [51]. But we are unaware of previous research discussing the possibility that prior anxiety disorders are especially likely to predispose to PTSD after an MVC occurs. One possibility is that the ubiquity of exposure to motor vehicles in the wake of a MVC makes avoidance especially difficult, with hyper-arousal in the face of re-exposure playing a more prominent role in post-traumatic reactions to MVCs than other traumatic experiences, possibly leading to prior anxiety disorders becoming especially important in promoting PTSD after MVCs.

Third, our finding that simulated sensitivity of post-MVC PTSD based on our model in an independent sample would be 32 % (more than six times the expected value) among the 5 % of respondents with highest predicted risk is broadly consistent with several recent more general studies showing that PTSD can be predicted significantly in the peri-traumatic period from information about pre-trauma risk factors, objective trauma characteristics, and early trauma responses [7, 52, 53]. We are also aware of one prior study that found good prediction accuracy of an index for subsequent PTSD among patients hospitalized after a severe injury [54], although not all such injuries were sustained in a MVC. It is noteworthy that it was until recently thought that the effect sizes of individual predictors in epidemiological models predicting PTSD were too low and inconsistent to be clinically useful in targeting people for preventive interventions [55], making it necessary to use assessment tools in the aftermath of trauma that focused on current symptoms rather than risk factors [56]. The recent studies cited above demonstrate clearly, though, that predictions based on multivariate models can be quite strong even when the coefficients for individual predictors are modest. Whether this prediction strength is high enough to be used in targeting preventive interventions is a more complex question that is beyond the scope of this paper, as such a determination requires the consideration not only of sensitivity but also of positive predictive value, the costs of treatment, treatment effectiveness, and the valuation of reductions in disability-adjustment life years associated with successful treatment.

It is important to note in this context that the development of a practical model to predict MVC-related PTSD for targeting preventive intervention would require a much larger sample than the one examined in the current report. Such a sample should be prospective, should include data collected in the immediate aftermath of MVCs, and should follow participants over time to determine which of them developed PTSD. A richer set of predictors than those considered in the current report should be included in the baseline assessment. Machine learning methods should be used to develop the prediction model in order to maximize out-of-sample performance [7]. Given the growing literature on the effectiveness of pharmacological and cognitive-behavioural interventions in individuals in the immediate aftermath of trauma [57–60], including interventions that have specifically been undertaken in survivors of MVCs [2, 61], our preliminary results suggest that the development of such an optimal prediction model might be of considerable value.

## Conclusions

This paper provides the first cross-national data on prevalence and predictors of MVC-related PTSD. Across the globe, PTSD is a relatively rare outcome of life-threatening MVCs. Further, significant predictors of PTSD after MVC are broadly consistent with previous work. Finally, a substantial minority of PTSD cases occur among the relatively small proportion of people with highest predicted risk; this raises the question whether MVC-related PTSD could be reduced with preventive interventions targeted to high-risk survivors.

## Abbreviations

AOO, age-of-onset; AUC, area under the curve; BPD, BP-I, BP-II, broadly-defined bipolar disorder, bipolar disorder I, bipolar disorder II; DSM-IV, Diagnostic and Statistical Manual of Mental Disorders IV; ICD-10, International Statistical Classification of Diseases and Related Health Problems, 10th revision; MVC, motor vehicle collision; OR, odds ratio; PTSD, post-traumatic stress disorder

## Additional files

Additional file 1: Table S1. World Mental Health sample characteristics by World Bank income categories. **Table S2.** The distribution of lifetime MVCs and DSM-IV/CIDI PTSD associated with MVCs in the participating World Mental Health surveys. **Table S3.** Prevalence of PTSD, by selected demographic and collision-specific characteristics, weighted analysis (*n* = 649). **Table S4.** Associations of individual prior traumatic events (TE’s) with DSM-IV/CIDI PTSD after randomly selected motor vehicle collisions in the total sample (*n* = 649). **Table S5.** Associations of childhood adversities (CAs) with DSM-IV/CIDI PTSD after randomly selected motor vehicle collisions in the total sample (*n* = 649). **Table S6.** Associations of prior DSM-IV/CIDI mental disorders with DSM-IV/CIDI PTSD after randomly selected motor vehicle collisions in the total sample (*n* = 649). (DOCX 74 kb) Additional file 2: Study Institutional Review Boards and Consent Features across WMH Survey Initiative. Names of study Institutional Review Boards and features of consent across the WMH Survey Initiative. (XLS 24 kb)
