# Supplementary material for: Post-traumatic stress disorder associated with life-threatening motor vehicle collisions in the WHO World Mental Health Surveys
Source: BMC Psychiatry. 2016 Jul 22;16:257. doi: 10.1186/s12888-016-0957-8 (PMC4957291; doi:10.1186/s12888-016-0957-8)
Supplement: Additional file 1: Table S1. — World Mental Health sample characteristics by World Bank income categories. Table S2. The distribution of lifetime MVCs and DSM-IV/CIDI PTSD associated with MVCs in the participating World Mental Health surveys. Table S3. Prevalence of PTSD, by selected demographic and collision-specific characteristics, weighted analysis (n = 649). Table S4. Associations of individual prior traumatic events (TE’s) with DSM-IV/CIDI PTSD after randomly selected motor vehicle collisions in the total sample (n = 649). Table S5. Associations of childhood adversities (CAs) with DSM-IV/CIDI PTSD after randomly selected motor vehicle collisions in the total sample (n = 649). Table S6. Associations of prior DSM-IV/CIDI mental disorders with DSM-IV/CIDI PTSD after randomly selected motor vehicle collisions in the total sample (n = 649). (DOCX 74 kb) [file 12888_2016_957_MOESM1_ESM.docx]

| **Appendix Table 1. World Mental Health sample characteristics by World Bank income categories*^1^*** | | |  |  |  |  | |  |  | |
| --- | --- | --- | --- | --- | --- | --- | --- | --- | --- | --- |
|  |  |  |  |  |  | **Sample Sizes** | | | | |
| **Country by income category** | **Survey*^2^*** | **Sample characteristics*^3^*** | **Field dates** | **Age range** | **Response rate*^4^*** | **Part 1** | **Part 2** | **Reported any traumatic experience** | **Study sample for MVC (excludes pedestrians, passerbys, and cyclists)** | |
| **I. High-income countries** |  |  |  |  |  |  |  |  |  | |
| Germany | ESEMeD | Nationally representative. | 2002-3 | 19-95 | 57.8 | 3,555 | 1,323 | 928 | 20 | |
| Israel | NHS | Nationally representative. | 2002-4 | 21-98 | 72.6 | 4,859 | 4,859 | 3,679 | 35 | |
| Spain | ESEMeD | Nationally representative. | 2001-2 | 18-98 | 78.6 | 5,473 | 2,121 | 1,284 | 58 | |
| Spain - Murcia | PEGASUS- Murcia | Murcia region. | 2010-12 | 18-96 | 67.4 | 2,621 | 1,459 | 942 | 39 | |
| United States | NCS-R | Nationally representative. | 2002-3 | 18-99 | 70.9 | 9,282 | 5,692 | 4,954 | 168 | |
| **TOTAL** |  |  |  |  | **70.1** | **(25,790)** | **(15,454)** | **(11,787)** | **(320)** | |
| **II. Low and middle-income countries** | |  |  |  |  |  |  |  |  | |
| Bulgaria | NSHS | Nationally representative. | 2003-7 | 18-98 | 72.0 | 5,318 | 2,233 | 776 | 31 | |
| Colombia – Medellin^5^ | MMHHS | Medellin metropolitan area | 2011-12 | 19-65 | 97.2 | 3,261 | 1,673 | 1,387 | 52 | |
| Lebanon | LEBANON | Nationally representative. | 2002-3 | 18-94 | 70.0 | 2,857 | 1,031 | 873 | 17 | |
| Mexico | M-NCS | All urban areas of the country^6^ | 2001-2 | 18-65 | 76.6 | 5,782 | 2,362 | 1,818 | 42 | |
| Peru | EMSMP | Nationally representative. | 2004-5 | 18-65 | 90.2 | 3,930 | 1,801 | 1,530 | 34 | |
| Romania | RMHS | Nationally representative. | 2005-6 | 18-96 | 70.9 | 2,357 | 2,357 | 997 | 63 | |
| South Africa^5^ | SASH | Nationally representative. | 2003-4 | 18-92 | 87.1 | 4,315 | 4,315 | 3,085 | 52 | |
| Ukraine^5^ | CMDPSD | Nationally representative. | 2002 | 18-91 | 78.3 | 4,725 | 1,720 | 1,545 | 38 | |
| **TOTAL** |  |  |  |  | **79.3** | **(32,545)** | **(17,492)** | **(12,011)** | **(329)** | |
| **III. TOTAL** |  |  |  |  | **75.0** | **(58,335)** | **(32,946)** | **(23,798)** | **(649)** | |
| *^1^*The World Bank (2012) Data. Accessed May 12, 2012 at: <http://data.worldbank.org/country>. Some of the World Mental Health countries have moved into new income categories since the surveys were conducted. The income groupings above reflect the status of each country at the time of data collection. The current income category of each country is available at the preceding URL.  *^2^*EMSMP (La Encuesta Mundial de Salud Mental en el Peru); CMDPSD (Comorbid Mental Disorders during Periods of Social Disruption); NSHS (Bulgaria National Survey of Health and Stress); MMHHS (Medellín Mental Health Household Study); LEBANON (Lebanese Evaluation of the Burden of Ailments and Needs of the Nation); M-NCS (The Mexico National Comorbidity Survey); RMHS (Romania Mental Health Survey); SASH (South Africa Health Survey); ESEMeD (The European Study Of The Epidemiology Of Mental Disorders); NHS (Israel National Health Survey); PEGASUS-Murcia (Psychiatric Enquiry to General Population in Southeast Spain-Murcia);NCS-R (The US National Comorbidity Survey Replication).  *^3^*Most World Mental Health surveys are based on stratified multistage clustered area probability household samples in which samples of areas equivalent to counties or municipalities in the US were selected in the first stage followed by one or more subsequent stages of geographic sampling (e.g., towns within counties, blocks within towns, households within blocks) to arrive at a sample of households, in each of which a listing of household members was created and one or two people were selected from this listing to be interviewed. No substitution was allowed when the originally sampled household resident could not be interviewed. These household samples were selected from Census area data in all countries used in this sample. Germany's sample used municipal resident registries to select respondents without listing households. 10 of the 13 surveys are based on nationally representative household samples.  *^4^*The response rate is calculated as the ratio of the number of households in which an interview was completed to the number of households originally sampled, excluding from the denominator households known not to be eligible either because of being vacant at the time of initial contact or because the residents were unable to speak the designated languages of the survey. The weighted average response rate is 75.0%.  *^5^*For the purposes of cross-national comparisons we limit the sample to those 18+.  ^6^Approximately 75% of the total national population | | | | | | | | | |  |

| **Appendix Table 2 The distribution of lifetime MVCs and DSM-IV/CIDI PTSD associated with MVCs in the participating World Mental Health** **surveys^1^** | | | | | | | | | | | |  |  |
| --- | --- | --- | --- | --- | --- | --- | --- | --- | --- | --- | --- | --- | --- |
|  | **% of respondents with MVC** | **(SE)** | **Mean # of occurrences /Any** | **(SE)** | **Frequency of MVC per 100 population** | **(SE)** | **MVCs as a % of all traumatic experiences in the population** | **(SE)** | **Probability of PTSD given a random MVC** | **(SE)** | **Number of lifetime cases of PTSD associated with MVC per 100 people in population** | **% of all PTSD cases in the population associated with MVC** | **(SE)** |
| **I. High-income countries** |  |  |  |  |  |  |  |  |  |  |  |  |  |
| Germany | 9.1 | (1.1) | 1.1 | (0.0) | 10.4 | (1.3) | 3.8 | (0.6) | 0.9 | (0.9) | 0.1 | 1.7 | (0.6) |
| Israel | 11.6 | (0.4) | 1.3 | (0.0) | 15.2 | (0.7) | 4.7 | (0.2) | 4.1 | (2.5) | 0.6 | 10.1 | (6.5) |
| Spain | 14.1 | (1.2) | 1.3 | (0.1) | 18.3 | (1.8) | 12.3 | (1.1) | 0.9 | (0.7) | 0.2 | 4.6 | (3.5) |
| Spain – Murcia | 11.9 | (0.7) | 1.3 | (0.1) | 15.0 | (1.7) | 9.6 | (1.2) | 0.2 | (0.2) | 0.0 | 0.7 | (0.1) |
| United States | 19.2 | (0.9) | 1.5 | (0.0) | 29.6 | (1.4) | 6.0 | (0.2) | 3.8 | (1.9) | 1.1 | 5.0 | (2.8) |
| Total | 14.6 | (0.4) | 1.4 | (0.0) | 20.5 | (0.7) | 6.0 | (0.2) | 3.1 | (1.2) | 0.6 | 5.4 | (2.2) |
| **II. Low and middle-income countries** | |  |  |  |  |  |  |  |  |  |  |  |  |
| Bulgaria | 6.9 | (0.9) | 1.4 | (0.1) | 9.5 | (1.3) | 12.8 | (1.4) | 5.7 | (2.0) | 0.5 | 7.9 | (2.2) |
| Colombia – Medellin | 18.4 | (1.7) | 1.4 | (0.1) | 26.2 | (3.3) | 6.5 | (0.6) | 1.5 | (1.1) | 0.4 | 2.0 | (0.5) |
| Lebanon | 12.8 | (1.4) | 1.3 | (0.1) | 16.9 | (2.0) | 4.4 | (0.5) | 0.1 | (0.1) | 0.0 | 0.2 | (0.1) |
| Mexico | 15.3 | (1.2) | 1.3 | (0.0) | 19.5 | (1.6) | 6.8 | (0.5) | 0.1 | (0.1) | 0.0 | 2.0 | (1.1) |
| Peru | 20.1 | (1.2) | 1.4 | (0.0) | 27.3 | (1.4) | 7.4 | (0.4) | 0.0 | (0.0) | -- | -- | -- |
| Romania | 9.3 | (0.6) | 1.4 | (0.1) | 13.4 | (1.2) | 10.2 | (0.8) | 1.6 | (1.6) | 0.2 | 3.6 | (0.9) |
| South Africa | 13.2 | (0.6) | 1.2 | (0.0) | 16.0 | (0.9) | 5.1 | (0.3) | 5.4 | (3.7) | 0.9 | 7.4 | (5.5) |
| Ukraine | 21.1 | (1.3) | 1.3 | (0.0) | 27.2 | (1.9) | 7.3 | (0.6) | 1.4 | (1.2) | 0.4 | 3.6 | (3.1) |
| Total | 14.4 | (0.4) | 1.3 | (0.0) | 18.6 | (0.6) | 6.6 | (0.2) | 2.1 | (0.8) | 0.4 | 4.7 | (1.8) |
| **III. Total** | 14.3 | (0.3) | 1.4 | (0.0) | 19.5 | (0.5) | 6.3 | (0.1) | 2.5 | (0.7) | 0.5 | 5.0 | (1.5) |
|  |  |  |  |  |  |  |  |  |  |  |  |  |  |

^1^The surveys considered here are limited to the subset of World Mental Health surveys that obtained information about traumatic experience characteristics associated with one randomly selected lifetime traumatic experience for each respondent and in which a sufficient number of respondents with a randomly selected MVC was included for at least one such respondent to have met DSM-IV/CIDI criteria for PTSD associated with that MVC. World Mental Health surveys that had too few randomly selected MVCs (numbers of such cases are reported in parentheses) for any to meet criteria for PTSD included those Brazil (23), Colombia (25), Japan (25), Northern Ireland (25), Belgium (13), France (34), Italy (46), Netherlands(9).

| **Appendix Table 3. Prevalence of PTSD, by selected demographic and collision-specific characteristics, weighted analysis (n=649)** | | | | | | | |
| --- | --- | --- | --- | --- | --- | --- | --- |
|  | **Distribution of PTSD** | | | |  | **Sample Characteristics** | |
|  | **% PTSD** | **(95% CI)** | **(SE)** | **(n)** |  | **%** | **(SE)** |
| Age at collision |  |  |  |  |  |  |  |
| Children (1-12) | 0.0 | (0.0-4.7) | -- | (43) |  | 11.7 | (3.3) |
| Adolescents (13-17) | 0.8 | (0.0-2.0) | (0.6) | (99) |  | 15.8 | (3.2) |
| Young adult (18-29) | 2.9 | (0.3-5.4) | (1.3) | (271) |  | 42.8 | (4.1) |
| Middle age (30-44) | 3.9 | (0.3-7.5) | (1.8) | (156) |  | 24.7 | (4.2) |
| Older (45+) | 10.5 | (2.0-19.0) | (4.3) | (80) |  | 5.0 | (0.9) |
| χ^2^_4_ | 6.7 | p=.16 |  | |  |  |  |
| Gender |  |  |  |  |  |  |  |
| Female | 4.1 | (1.1-7.0) | (1.5) | (331) |  | 40.9 | (4.2) |
| Male | 2.0 | (0.5-3.5) | (0.8) | (318) |  | 59.1 | (4.2) |
| χ^2^_1_ | 1.6 | p=.21 |  | |  |  |  |
| Education |  |  |  |  |  |  |  |
| Low | 2.7 | (0.5-4.9) | (1.1) | (161) |  | 27.9 | (4.1) |
| Low average | 5.3 | (0.0-10.5) | (2.7) | (157) |  | 20.5 | (3.0) |
| High average | 2.2 | (0.1-4.3) | (1.1) | (224) |  | 38.1 | (4.2) |
| High | 1.2 | (0.0-2.9) | (0.8) | (107) |  | 13.5 | (2.5) |
| χ^2^_3_ | 4.6 | p=.21 |  | |  |  |  |
| Marital status |  |  |  |  |  |  |  |
| Never married | 1.9 | (0.6-3.2) | (0.7) | (404) |  | 66.9 | (4.2) |
| Currently married | 5.0 | (0.3-9.6) | (2.4) | (183) |  | 25.1 | (4.1) |
| Previously married | 4.3 | (0.0-9.9) | (2.8) | (62) |  | 8.0 | (2.1) |
| χ^2^_2_ | 10.1 | p=.006 |  | |  |  |  |
| Role of respondent |  |  |  |  |  |  |  |
| Passenger | 2.3 | (0.7-4.0) | (0.8) | (364) |  | 54.1 | (4.2) |
| Driver | 3.5 | (0.8-6.1) | (1.3) | (285) |  | 45.9 | (4.2) |
| χ^2^_1_ | 1.0 | p=.33 |  | |  |  |  |
| Who was at fault |  |  |  |  |  |  |  |
| Respondent | 1.3 | (0.0-2.9) | (0.8) | (116) |  | 20.6 | (3.3) |
| Someone else | 3.2 | (1.0-5.4) | (1.1) | (404) |  | 62.8 | (4.0) |
| No fault/weather/missing | 3.4 | (0.2-6.7) | (1.6) | (129) |  | 16.6 | (2.9) |
| χ^2^_2_ | 1.5 | p=.48 |  | |  |  |  |
| Anyone killed? |  |  |  |  |  |  |  |
| No | 2.4 | (1.0-3.8) | (0.6) | (609) |  | 95.1 | (1.7) |
| Yes | 11.6 | (0.0-26.1) | (7.4) | (40) |  | 4.9 | (1.7) |
| χ^2^_1_ | 6.8 | p=.009 |  | |  |  |  |
| Respondent injured? |  |  |  |  |  |  |  |
| No | 1.6 | (0.5-2.6) | (0.5) | (415) |  | 68.6 | (3.9) |
| Yes | 5.6 | (1.4-9.8) | (2.2) | (234) |  | 31.4 | (3.9) |
| χ^2^_1_ | 8.3 | p=.004 |  | |  |  |  |
| Someone else injured? |  |  |  |  |  |  |  |
| No | 2.2 | (0.7-3.8) | (0.8) | (522) |  | 79.5 | (3.7) |
| Yes | 5.2 | (0.8-9.6) | (2.2) | (127) |  | 20.5 | (3.7) |
| χ^2^_1_ | 4.3 | p=.038 |  | |  |  |  |
| Prior collisions |  |  |  |  |  |  |  |
| 0 | 2.3 | (1.0-3.6) | (0.6) | (604) |  | 80.7 | (3.9) |
| 1 | 1.4 | (0.0-3.2) | (1.0) | (32) |  | 13.0 | (3.5) |
| 2+ | 12.6 | (0.0-30.0) | (8.9) | (13) |  | 6.3 | (2.3) |
| χ^2^_2_ | 7.9 | p=.019 |  | |  |  |  |
| Prior trauma types |  |  |  |  |  |  |  |
| 0 | 2.3 | (0.5-4.2) | (0.9) | (428) |  | 44.4 | (4.1) |
| 1 | 1.7 | (0.1-3.2) | (0.8) | (111) |  | 32.3 | (4.6) |
| 2 | 5.5 | (0.6-10.4) | (2.5) | (110) |  | 23.2 | (3.3) |
| χ^2^_2_ | 6.8 | p=.033 |  |  |  |  |  |
| Number of childhood adversities |  |  |  |  |  |  |  |
| 0 | 2.1 | (0.8-3.4) | (0.7) | (502) |  | 73.1 | (3.6) |
| 1 | 2.3 | (0.0-5.4) | (1.6) | (87) |  | 12.2 | (2.3) |
| 2 | 7.0 | (0.0-14.5) | (3.8) | (60) |  | 14.7 | (3.2) |
| χ^2^_3_ | 7.9 | p=.019 |  | |  |  |  |
| Number of anxiety disorders |  |  |  |  |  |  |  |
| 0 | 1.5 | (0.4-2.6) | (0.6) | (525) |  | 82.4 | (3.0) |
| 1 | 5.0 | (0.6-9.3) | (2.2) | (91) |  | 10.8 | (2.1) |
| 2 | 4.0 | (0.0-10.7) | (3.4) | (18) |  | 4.2 | (2.1) |
| 3+ | 33.8 | (1.4-66.3) | (16.5) | (15) |  | 2.6 | (0.9) |
| χ^2^_3_ | 23.5 | p<.001 |  | |  |  |  |
| Total | 2.8 | (1.3-4.3) | (0.8) | (649) |  | -- | -- |
|  |  |  |  |  |  |  |  |

The analysis is limited to respondents who were either drivers or passengers in motor vehicles. All results are based on weighted data. See the text for a description of the weighting procedures. Confidence intervals for subgroups in which weighted prevalence estimates were less than two times the design-based standard errors of these estimates were estimated using the Wilson score interval method [30]..

| **Appendix Table 4. Associations of individual prior traumatic events (TE’s) with DSM-IV/CIDI PTSD after randomly selected motor vehicle collisions in the total sample (n=649)** | | | | | | | | |
| --- | --- | --- | --- | --- | --- | --- | --- | --- |
|  | **Bivariate** | |  | **Multivariate Model 1** | |  | **Multivariate Model 2** | |
|  | **OR** | **(95% CI)** |  | **OR** | **(95% CI)** |  | **OR** | **(95% CI)** |
| I. Exposure to sectarian violence |  |  |  |  |  |  |  |  |
| Relief worker in war zone | -- | -- |  | -- | -- |  | -- | -- |
| Civilian in war zone | 0.9 | (0.3-3.0) |  | 0.7 | (0.1-3.5) |  | -- | -- |
| Civilian in region of terror | 0.9 | (0.1-6.8) |  | 1.2 | (0.3-5.8) |  | -- | -- |
| Refugee | 1.4 | (0.3-7.4) |  | 0.3 | (0.1-2.1) |  | -- | -- |
| Kidnapped | 0.0 | (0.0-0.0) |  | -- | -- |  | -- | -- |
| Any | 1.0 | (0.5-2.1) |  | -- | -- |  | 0.8 | (0.4-1.6) |
| II. Participation in sectarian violence |  |  |  |  |  |  |  |  |
| Combat experience | 0.0 | (0.0-0.0) |  | -- | -- |  | -- | -- |
| Witnessed death | 4.5* | (1.4-14.6) |  | 0.7 | (0.3-2.0) |  | -- | -- |
| Accidentally cause injury/death | 0.0 | (0.0-0.0) |  | -- | -- |  | -- | -- |
| Purposefully injured/killed someone | 0.0 | (0.0-0.0) |  | -- | -- |  | -- | -- |
| Saw atrocities | 1.0 | (0.1-12.3) |  | 2.5 | (0.4-17.8) |  | -- | -- |
| Any | 3.7 | (1.1-12.5) |  | -- | -- |  | 2.1 | (0.7-5.9) |
| III. Interpersonal violence |  |  |  |  |  |  |  |  |
| Beaten by caregiver | 5.3 | (0.7-37.9) |  | 0.6 | (0.1-3.0) |  | -- | -- |
| Beaten by someone else | 0.0 | (0.0-0.0) |  | -- | -- |  | -- | -- |
| Witnessed physical fight at home | 35.6* | (8.2-154.4) |  | 54.1* | (9.5-309.0) |  | -- | -- |
| Any | 8.2* | (1.7-40.3) |  | -- | -- |  | 5.4* | (1.4-21.6) |
| IV. Exposure to sexual violence |  |  |  |  |  |  |  |  |
| Beaten by spouse/partner | 1.5 | (0.0-44.4) |  | 2.3 | (0.2-27.6) |  | -- | -- |
| Raped | 10.4* | (2.0-55.3) |  | 1.9 | (0.4-8.4) |  | -- | -- |
| Sexually assaulted | 0.6 | (0.1-5.4) |  | 0.1 | (0.0-0.2) |  | -- | -- |
| Stalked | 0.0 | (0.0-0.0) |  | -- | -- |  | -- | -- |
| Traumatic event to loved one | 10.5* | (1.6-69.1) |  | 1.4 | (0.0-271.3) |  | -- | -- |
| Some other event | 0.0 | (0.0-0.0) |  | -- | -- |  | -- | -- |
| Private event | 2.8 | (0.2-35.2) |  | 57.0* | (2.0-1632) |  | -- | -- |
| Any | 2.2 | (0.6-8.1) |  | -- | -- |  | 1.3 | (0.4-3.9) |
| V. Accident/injuries |  |  |  |  |  |  |  |  |
| Toxic chemical exposure | 0.5 | (0.0-10.4) |  | 0.1 | (0.0-1.3) |  | -- | -- |
| Automobile collision | 3.9* | (1.6-9.6) |  | 4.1* | (1.7-9.5) |  | -- | -- |
| Other life threatening accident | 0.3 | (0.0-5.7) |  | 0.2 | (0.0-3.5) |  | -- | -- |
| Natural disaster | 7.4* | (1.7-32.2) |  | 3.7 | (0.9-14.8) |  | -- | -- |
| Life-threatening illness | 9.3* | (2.6-33.7) |  | 9.7* | (2.0-48.0) |  | -- | -- |
| Child with serious illness | 0.6 | (0.0-12.7) |  | 0.1 | (0.0-21.1) |  | -- | -- |
| Any | 1.4 | (0.6-3.5) |  | -- | -- |  | 1.0 | (0.3-2.8) |
| VI. Other trauma |  |  |  |  |  |  |  |  |
| Man-made disaster | 1.0 | (0.1-9.6) |  | 0.8 | (0.0-36.7) |  | -- | -- |
| Mugged or threatened with a weapon | 2.9 | (0.9-9.5) |  | 1.4 | (0.2-9.1) |  | -- | -- |
| Unexpected death of a loved one | 1.2 | (0.2-5.6) |  | 2.4 | (0.8-6.9) |  | -- | -- |
| Any | 1.5 | (0.3-7.4) |  | -- | -- |  | 1.5 | (0.4-5.9) |
| F_(21, 112), (6, 127)_ |  |  |  | 9.4* | p < .001 |  | 2.0 | p = .07 |
| F_(20, 113), (5, 128)_ |  |  |  | 9.4* | p < .001 |  | 1.5 | p = .19 |
| VII. Numbers of prior traumatic experience types |  |  |  |  |  |  |  |  |
| Any | 1.2 | (0.6-2.4) |  | -- | -- |  | -- | -- |
| Exactly 1 | 0.7 | (0.2-2.3) |  | -- | -- |  | -- | -- |
| Exactly 2 | 0.5 | (0.1-1.9) |  | -- | -- |  | -- | -- |
| 3+ | 3.4 | (1.4-6.0) |  | -- | -- |  | -- | -- |
| F_(3, 130)_ | 3.8* | p = .01 |  |  |  |  |  |  |
|  |  |  |  |  |  |  |  |  |

^1^All models controlled for the predictors in Model 2 in the main text. Multivariate models excluded prior traumatic experiences with no case of PTSD after the randomly selected MVC. Models were based on weighted data. See the text for a description of weighting procedures.

^2^Joint significance of the 3 coefficients associated with number of traumatic experience types (p=.21)

^3^Joint significance of the 20 coefficients other than the one associated with prior MVCs (p=.90)

^4^Joint significance of the 6 coefficients associated with any traumatic experience in the 6 row categories (p=.33). These categories were defined based on the results of an exploratory factor analysis described in more detail elsewhere [48].

| **Appendix Table 5. Associations of childhood adversities (CAs) with DSM-IV/CIDI PTSD after randomly selected motor vehicle collisions in the total sample (n=649)** | | | | | | | | |
| --- | --- | --- | --- | --- | --- | --- | --- | --- |
|  | **Bivariate** | |  | **Multivariate Model 1** | |  | **Multivariate Model 2** | |
|  | **OR** | **(95% CI)** |  | **OR** | **(95% CI)** |  | **OR** | **(95% CI)** |
| I. Maladaptive family functioning (MFF) childhood adversity (CA) | | |  |  |  |  |  |  |
| Parental mental illness | 1.7 | (0.4-7.7) |  | 0.5 | (0.2-1.6) |  | 0.5 | (0.2-1.6) |
| Parental substance misuse | 3.3 | (0.6-18.4) |  | 1.9 | (0.3-10.4) |  | 2.1 | (0.4-11.7) |
| Parental criminality | 10.6* | (2.5-45.0) |  | 4.0* | (1.7-9.1) |  | 2.6 | (0.7-8.7) |
| Family violence | 14.2* | (3.5-57.2) |  | 5.4* | (1.1-25.3) |  | 4.5 | (0.9-22.6) |
| Physical abuse | 6.4* | (1.3-32.1) |  | 0.4 | (0.1-1.4) |  | 0.3* | (0.1-0.9) |
| Sexual abuse | 12.8* | (2.4-68.5) |  | 1.4 | (0.3-5.9) |  | 1.3 | (0.3-5.3) |
| Neglect | 20.9* | (4.0-110.4) |  | 9.5* | (1.6-55.6) |  | 7.7* | (1.1-55.0) |
| F_(7, 126)_ |  |  |  | 7.5* | p <.001 |  | 2.4* | p =.02 |
| F_(6, 127)_ |  |  |  | 3.0* | p =.01 |  | 2.5* | p =.03 |
| II. Other CA |  |  |  |  |  |  |  |  |
| Parental death | 0.8 | (0.2-3.6) |  | 1.1 | (0.3-4.2) |  | 0.8 | (0.1-4.3) |
| Parental divorce | 6.0* | (1.3-28.2) |  | 0.7 | (0.1-5.1) |  | 0.4 | (0.0-4.5) |
| Other parental loss | 0.0 | (0.0-0.0) |  | -- | -- |  | -- | -- |
| Serious physical illness | 2.4 | (0.5-11.7) |  | 3.1 | (0.8-12.0) |  | 2.6 | (0.6-11.5) |
| Family economic adversity | 0.6 | (0.1-5.6) |  | 0.6 | (0.1-3.0) |  | 0.3 | (0.0-2.2) |
| F_(4, 129)_ |  |  |  | 1.5 | p =.20 |  | 1.9 | p =.11 |
| F_(3, 130)_ |  |  |  | 2.0 | p =.12 |  | 2.5 | p =.06 |
| F_(11, 122)_ |  |  |  | 6.1* | p <.001 |  | 3.8* | p <.001 |
| F_(10, 123)_ |  |  |  | 2.8* | P = .004 |  | 2.4* | p = .01 |
| III. Number of MFF CAs |  |  |  |  |  |  |  |  |
| Exactly 1 | 1.5 | (0.4-5.8) |  | -- | -- |  | -- | -- |
| 2+ | 12.8* | (3.4-47.5) |  | -- | -- |  | 1.8 | (0.2-15.6) |
| F_(2, 131)_ | 7.4* | p=.001 |  |  |  |  | 0.1 | p=.76 |
| IV. Number of other CAs |  |  |  |  |  |  |  |  |
| Exactly 1 | 0.4 | (0.1-1.3) |  | -- | -- |  | -- | -- |
| 2+ | 4.3 | (1.0-18.1) |  | -- | -- |  | 4.5 | (0.5-37.5) |
| F_(2, 131)_ | 4.5* | p=.01 |  |  |  |  | 0.6 | p=.42 |
|  |  |  |  |  |  |  |  |  |

^*^Significant at the .05 level, two-sided design-based test

^1^All models controlled for the predictors in Model 2 in the main text. Multivariate models excluded CAs associated with no case of PTSD after the randomly selected MVC. Models were based on weighted data. See the text for a description of weighting procedures.

^2^Joint significance of all the CA measures in the set above the χ2 test

^3^Significance of differences in ORs across all the CA measures in the set above the F test

^4^Joint significance of the 11 CA measures

| **Appendix Table 6. Associations of prior DSM-IV/CIDI mental disorders with DSM-IV/CIDI PTSD after randomly selected motor vehicle collisions in the total sample (n=649)^1^** | | | | | | | | | | | | | | |
| --- | --- | --- | --- | --- | --- | --- | --- | --- | --- | --- | --- | --- | --- | --- |
|  | **Bivariate** | |  | **Multivariate Model 1** | |  | **Multivariate Model 2** | |  | **Multivariate Model 3** | |  | **Multivariate Model 4** | |
|  | **OR** | **(95% CI)** |  | **OR** | **(95% CI)** |  | **OR** | **(95% CI)** |  | **OR** | **(95% CI)** |  | **OR** | **(95% CI)** |
| I. Mood disorders |  |  |  |  |  |  |  |  |  |  |  |  |  |  |
| MDE or dysthymia | 3.1* | (1.3-7.2) |  | 0.8 | (0.4-1.7) |  | 0.8 | (0.4-1.7) |  | -- | -- |  | -- | -- |
| BPD | 1.8 | (0.1-26.0) |  | 16.6* | (2.0-140.6) |  | 28.4* | (2.2-358.8) |  | -- | -- |  | -- | -- |
| Any mood | 2.4* | (1.0-5.5) |  | . | -- |  | -- | -- |  | 0.6 | (0.2-1.6) |  | 0.7 | (0.3-1.8) |
| II. Anxiety disorders |  |  |  |  |  |  |  |  |  |  |  |  |  |  |
| Agor/panic | 0.1* | (0.0-0.4) |  | 0.0* | (0.0-0.0) |  | 0.0* | (0.0-0.0) |  | -- | -- |  | -- | -- |
| GAD | 8.2* | (2.2-30.4) |  | 2.1 | (0.4-12.3) |  | 2.5 | (0.4-16.3) |  | -- | -- |  | -- | -- |
| PTSD | 15.9* | (4.9-52.2) |  | 19.2* | (3.4-108.8) |  | 33.5* | (3.8-293.4) |  | -- | -- |  | 1.4 | (0.2-9.0) |
| Social phobia | 53.6* | (17.2-167.2) |  | 141.6* | (15.7-1277.0) |  | 263.4* | (18.6-3722.0) |  | -- | -- |  | -- | -- |
| Specific phobia | 3.6* | (1.0-12.3) |  | 0.3 | (0.0-1.9) |  | 1.4 | (0.3-5.9) |  | -- | -- |  | -- | -- |
| SAD | 3.5 | (0.8-15.3) |  | 0.4 | (0.0-2.8) |  | 1.7 | (0.2-18.0) |  | -- | -- |  | -- | -- |
| Number | 4.7* | (2.6-8.7) |  | . | -- |  | -- | -- |  | 4.3* | (2.1-8.8) |  | 5.4* | (1.0-28.9) |
| III. Disruptive behavioral disorders | |  |  |  |  |  |  |  |  |  |  |  |  |  |
| ADHD | 9.4* | (2.8-31.7) |  | 0.0* | (0.0-0.3) |  | 0.1* | (0.0-0.8) |  | -- | -- |  | -- | -- |
| Conduct | 0.0* | (0.0-0.0) |  | - | - |  | - | - |  | -- | -- |  | -- | -- |
| IED | 5.3* | (1.0-27.0) |  | 0.6 | (0.1-3.3) |  | 0.7 | (0.1-3.8) |  | -- | -- |  | -- | -- |
| ODD | 16.2* | (5.3-50.0) |  | 138.0* | (13.6-1404.0) |  | 182.7* | (15.0-2223.0) |  | -- | -- |  | -- | -- |
| Number | 2.9* | (1.8-4.5) |  | . | -- |  | -- | -- |  | 1.7 | (0.8-3.3) |  | 2.4 | (0.8-7.3) |
| IV. Substance disorders |  |  |  |  |  |  |  |  |  |  |  |  |  |  |
| Drug | 0.0 | (0.0-0.0) |  | - | -- |  | -- | -- |  | -- | -- |  | -- | -- |
| Alcohol | 4.5* | (1.7-12.0) |  | 8.8* | (3.0-25.8) |  | 15.8* | (4.6-53.8) |  | -- | -- |  | -- | -- |
| Any | 3.9* | (1.5-10.4) |  |  | -- |  | -- | -- |  | 1.9 | (0.6-6.0) |  | 2.8 | (0.7-10.6) |
| F_(12, 121), (12, 121), (4, 129), (4, 129)_ |  |  |  | 4.5* | p<.001 |  | 2.5* | p=.01 |  | 6.1* | p<.001 |  | 1.8 | p=.13 |
| F_(12, 122), (11, 122), (3, 130), (3, 130)_ |  |  |  | 3.2* | p=.001 |  | 2.7* | p=.004 |  | 2.9 | p=.04 |  | 2.3 | p=.08 |
| V. Total |  |  |  |  |  |  |  |  |  |  |  |  |  |  |
| Any | 8.9* | (3.4-23.2) |  | -- | -- |  | -- | -- |  | -- | -- |  | -- | -- |
| Exactly 1 | 4.9* | (1.7-14.2) |  | -- | -- |  | -- | -- |  | -- | -- |  | -- | -- |
| Exactly 2 | 27.3* | (8.1-91.9) |  | -- | -- |  | 0.9 | (0.2-5.1) |  | -- | -- |  | 1.6 | (0.2-12.4) |
| 3+ | 35.0* | (10.3-118.6) |  | -- | -- |  | 0.0 | (0.0-1.1) |  | -- | -- |  | 0.1 | (0.0-7.7) |
| F_(3, 130), (2, 131), (2, 131)_ | 13.3* | p < .001 |  |  |  |  | 6.0* | p=.003 |  |  |  |  | 4.8* | p=.01 |
|  |  |  |  |  |  |  |  |  |  |  |  |  |  |  |

^*^Significant at the .05 level, two-sided test.

^1^All models controlled for the predictors in Model 3 in the main text. Multivariate models excluded disorders associated with no case of PTSD after the randomly selected MVC. Models were based on weighted data. See the text for a description of weighting procedures.

^2^Joint significance of the 3 dummy variables for number of prior disorders.

^3^Joint significance of all 14 prior disorders.

^4^Significance of differences in ORs across all the 14 prior disorders.

^4^Joint significance of the 4 summary disorder type measures.
